# Supplementary material for: Unveiling the Bioactive Potential of the Invasive Jellyfish Phyllorhiza punctata Through Integrative Transcriptomic and Proteomic Analyses
Source: Biomolecules. 2025 Aug 4;15(8):1121. doi: 10.3390/biom15081121 (PMC12383608; doi:10.3390/biom15081121)
Supplement: Supplementary file 1 [file biomolecules-15-01121-s001.zip › Suplementary Material/FileS3/PEPFOLD4/XP_065054624_3880/clusters.html]

 PEP-FOLD clusters


Download archive of all models  here   

|  |  |  |
| --- | --- | --- |
| # | file | sOPEP |
| # cluster 1 - Size: 1 |  |  |
|  | XP\_065054624\_3880-model1 | -7.59195 |
| # cluster 2 - Size: 1 |  |  |
|  | XP\_065054624\_3880-model2 | -7.17559 |
| # cluster 3 - Size: 5 |  |  |
|  | XP\_065054624\_3880-model3 | -7.17208 |
|  | XP\_065054624\_3880-model3.1 | -6.40667 |
|  | XP\_065054624\_3880-model3.2 | -6.10794 |
|  | XP\_065054624\_3880-model3.3 | -5.57944 |
|  | XP\_065054624\_3880-model3.4 | -4.62522 |
| # cluster 4 - Size: 1 |  |  |
|  | XP\_065054624\_3880-model4 | -7.11141 |
| # cluster 5 - Size: 1 |  |  |
|  | XP\_065054624\_3880-model5 | -7.10187 |
| # cluster 6 - Size: 4 |  |  |
|  | XP\_065054624\_3880-model6 | -6.96725 |
|  | XP\_065054624\_3880-model6.1 | -6.24242 |
|  | XP\_065054624\_3880-model6.2 | -5.71006 |
|  | XP\_065054624\_3880-model6.3 | -5.01815 |
| # cluster 7 - Size: 11 |  |  |
|  | XP\_065054624\_3880-model7 | -6.91825 |
|  | XP\_065054624\_3880-model7.1 | -6.9174 |
|  | XP\_065054624\_3880-model7.2 | -6.63302 |
|  | XP\_065054624\_3880-model7.3 | -5.84266 |
|  | XP\_065054624\_3880-model7.4 | -5.73799 |
|  | XP\_065054624\_3880-model7.5 | -5.68939 |
|  | XP\_065054624\_3880-model7.6 | -5.55453 |
|  | XP\_065054624\_3880-model7.7 | -5.40503 |
|  | XP\_065054624\_3880-model7.8 | -5.28478 |
|  | XP\_065054624\_3880-model7.9 | -5.21565 |
|  | XP\_065054624\_3880-model7.10 | -5.20313 |
| # cluster 8 - Size: 7 |  |  |
|  | XP\_065054624\_3880-model8 | -6.84856 |
|  | XP\_065054624\_3880-model8.1 | -6.05933 |
|  | XP\_065054624\_3880-model8.2 | -5.8686 |
|  | XP\_065054624\_3880-model8.3 | -5.81349 |
|  | XP\_065054624\_3880-model8.4 | -5.44824 |
|  | XP\_065054624\_3880-model8.5 | -5.38467 |
|  | XP\_065054624\_3880-model8.6 | -4.83596 |
| # cluster 9 - Size: 3 |  |  |
|  | XP\_065054624\_3880-model9 | -6.71659 |
|  | XP\_065054624\_3880-model9.1 | -5.06673 |
|  | XP\_065054624\_3880-model9.2 | -4.66544 |
| # cluster 10 - Size: 2 |  |  |
|  | XP\_065054624\_3880-model10 | -6.65101 |
|  | XP\_065054624\_3880-model10.1 | -5.41316 |
| # cluster 11 - Size: 4 |  |  |
|  | XP\_065054624\_3880-model11 | -6.28091 |
|  | XP\_065054624\_3880-model11.1 | -5.52186 |
|  | XP\_065054624\_3880-model11.2 | -5.04852 |
|  | XP\_065054624\_3880-model11.3 | -4.64114 |
| # cluster 12 - Size: 4 |  |  |
|  | XP\_065054624\_3880-model12 | -5.69269 |
|  | XP\_065054624\_3880-model12.1 | -5.35541 |
|  | XP\_065054624\_3880-model12.2 | -5.27585 |
|  | XP\_065054624\_3880-model12.3 | -4.88485 |
| # cluster 13 - Size: 2 |  |  |
|  | XP\_065054624\_3880-model13 | -5.62465 |
|  | XP\_065054624\_3880-model13.1 | -4.83892 |
| # cluster 14 - Size: 2 |  |  |
|  | XP\_065054624\_3880-model14 | -5.59983 |
|  | XP\_065054624\_3880-model14.1 | -5.20476 |
| # cluster 15 - Size: 2 |  |  |
|  | XP\_065054624\_3880-model15 | -5.47063 |
|  | XP\_065054624\_3880-model15.1 | -5.06444 |
| # cluster 16 - Size: 6 |  |  |
|  | XP\_065054624\_3880-model16 | -5.44232 |
|  | XP\_065054624\_3880-model16.1 | -5.09186 |
|  | XP\_065054624\_3880-model16.2 | -5.06265 |
|  | XP\_065054624\_3880-model16.3 | -5.02347 |
|  | XP\_065054624\_3880-model16.4 | -4.93179 |
|  | XP\_065054624\_3880-model16.5 | -4.87048 |
| # cluster 17 - Size: 1 |  |  |
|  | XP\_065054624\_3880-model17 | -5.34048 |
| # cluster 18 - Size: 3 |  |  |
|  | XP\_065054624\_3880-model18 | -5.29684 |
|  | XP\_065054624\_3880-model18.1 | -5.14922 |
|  | XP\_065054624\_3880-model18.2 | -5.03849 |
| # cluster 19 - Size: 3 |  |  |
|  | XP\_065054624\_3880-model19 | -5.29628 |
|  | XP\_065054624\_3880-model19.1 | -5.06787 |
|  | XP\_065054624\_3880-model19.2 | -4.78326 |
| # cluster 20 - Size: 4 |  |  |
|  | XP\_065054624\_3880-model20 | -5.23671 |
|  | XP\_065054624\_3880-model20.1 | -4.95628 |
|  | XP\_065054624\_3880-model20.2 | -4.90415 |
|  | XP\_065054624\_3880-model20.3 | -4.56033 |
| # cluster 21 - Size: 5 |  |  |
|  | XP\_065054624\_3880-model21 | -5.2187 |
|  | XP\_065054624\_3880-model21.1 | -4.73694 |
|  | XP\_065054624\_3880-model21.2 | -4.71704 |
|  | XP\_065054624\_3880-model21.3 | -4.70863 |
|  | XP\_065054624\_3880-model21.4 | -4.61242 |
| # cluster 22 - Size: 3 |  |  |
|  | XP\_065054624\_3880-model22 | -5.12681 |
|  | XP\_065054624\_3880-model22.1 | -5.08366 |
|  | XP\_065054624\_3880-model22.2 | -4.93268 |
| # cluster 23 - Size: 2 |  |  |
|  | XP\_065054624\_3880-model23 | -5.08229 |
|  | XP\_065054624\_3880-model23.1 | -4.65821 |
| # cluster 24 - Size: 3 |  |  |
|  | XP\_065054624\_3880-model24 | -5.02656 |
|  | XP\_065054624\_3880-model24.1 | -4.36271 |
|  | XP\_065054624\_3880-model24.2 | -4.34725 |
| # cluster 25 - Size: 2 |  |  |
|  | XP\_065054624\_3880-model25 | -4.99895 |
|  | XP\_065054624\_3880-model25.1 | -4.43462 |
| # cluster 26 - Size: 1 |  |  |
|  | XP\_065054624\_3880-model26 | -4.99556 |
| # cluster 27 - Size: 2 |  |  |
|  | XP\_065054624\_3880-model27 | -4.98912 |
|  | XP\_065054624\_3880-model27.1 | -4.70704 |
| # cluster 28 - Size: 1 |  |  |
|  | XP\_065054624\_3880-model28 | -4.9131 |
| # cluster 29 - Size: 3 |  |  |
|  | XP\_065054624\_3880-model29 | -4.90854 |
|  | XP\_065054624\_3880-model29.1 | -4.63424 |
|  | XP\_065054624\_3880-model29.2 | -4.44313 |
| # cluster 30 - Size: 4 |  |  |
|  | XP\_065054624\_3880-model30 | -4.8173 |
|  | XP\_065054624\_3880-model30.1 | -4.80393 |
|  | XP\_065054624\_3880-model30.2 | -4.39181 |
|  | XP\_065054624\_3880-model30.3 | -4.38566 |
| # cluster 31 - Size: 1 |  |  |
|  | XP\_065054624\_3880-model31 | -4.71845 |
| # cluster 32 - Size: 1 |  |  |
|  | XP\_065054624\_3880-model32 | -4.60582 |
| # cluster 33 - Size: 1 |  |  |
|  | XP\_065054624\_3880-model33 | -4.571 |
| # cluster 34 - Size: 2 |  |  |
|  | XP\_065054624\_3880-model34 | -4.55095 |
|  | XP\_065054624\_3880-model34.1 | -4.30889 |
| # cluster 35 - Size: 1 |  |  |
|  | XP\_065054624\_3880-model35 | -4.2796 |
| # cluster 36 - Size: 1 |  |  |
|  | XP\_065054624\_3880-model36 | -4.26904 |
|  |  |  |
